# Polyaniline inside the pores of high surface area mesoporous silicon as composite electrode material for supercapacitors†

**DOI:** 10.1039/d2ra01829b

**Published:** 2022-06-10

**Authors:** Saima Nawaz, Yaqoob Khan, Shaimaa A. M. Abdelmohsen, Sadia Khalid, Emma M. Björk, Muhammad Asim Rasheed, M. Siddiq

**Affiliations:** Department of Chemistry, Quaid-i-Azam University Islamabad 45320 Pakistan m_sidiq12@yahoo.com +92 5190642147; Nanoscience and Technology Department, National Centre for Physics QAU Campus, Shahdra Valley Road Islamabad 45320 Pakistan yaqoob@ncp.edu.pk +92 512077389 +92 3455235423; Department of Physics, College of Science, Princess Nourah Bint Abdulrahman University P. O. Box 84428 Riyadh 11681 Saudi Arabia; Nanostructured Materials, Department of Physics, Chemistry and Biology (IFM), Linköping University SE-581 83 Linköping Sweden; Department of Physics and Applied Mathematics, Pakistan Institute of Engineering and Applied Sciences (PIEAS) Islamabad 45650 Pakistan

## Abstract

Mesoporous silicon (mSi) obtained by the magnesiothermic reduction of mesoporous silica was used to deposit polyaniline (PANI) in its pores, the composite was tested for its charge storage application for high performance supercapacitor electrodes. The mesoporous silica as confirmed by Small Angle X-ray Scattering (SAXS) has a Brunauer–Emmett–Teller (BET) surface area of 724 m^2^g^−1^ and mean pore size of 5 nm. After magnesiothermic reduction to mSi, the BET surface area is reduced to 348 m^2^g^−1^ but the mesoporousity is retained with a mean pore size of 10 nm. The BET surface area of mesoporous silicon is among the highest for porous silicon prepared/reduced from silica. *In situ* polymerization of PANI inside the pores of mSi was achieved by controlling the polymerization conditions. As a supercapacitor electrode, the mSi–PANI composite exhibits better charge storage performance as compared to pure PANI and mesoporous silica–PANI composite electrodes. Enhanced electrochemical performance of the mSi–PANI composite is attributed to the high surface mesoporous morphology of mSi with a network structure containing abundant mesopores enwrapped by an electrochemically permeable polyaniline matrix.

## Abstract 2

Magnesiothermic reduction was used to reduce mesoporous silica to mesoporous silicon which can host a variety of materials such as polyaniline and has potential to be used in supercapacitors.

## Graphical abstract

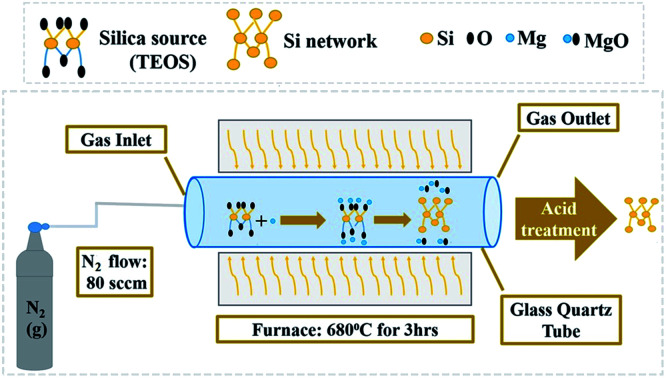

## Introduction

Supercapacitors are the next generation of energy storage devices designed to traverse the gap between capacitors and batteries. Supercapacitors have an immense scope in the energy sector to replace batteries due to their fast charging–discharging rate for storing energy with high power density, intermediate specific energy and excellent long cycle life. They are a stronger candidate for the working territory where devices demand urgent power to be sustained for a short duration *e.g.*, in electric vehicles. Based on the mechanism of charge storage, supercapacitors are categorized in two types *i.e.*, pseudo capacitors and electric double layer capacitors (EDLCs). Energy is stored in pseudocapacitors *via* fast reversible redox reactions and this mechanism is seen in electrically conductive polymers and transition metal oxides. EDLCs have a storage mechanism of ion absorption at the interface of the electrolyte and the electrical double layer at the electrode. Carbon-based active materials comprising high surface area fall in the category of EDLCs. Pseudocapacitors exhibit higher specific capacitance as compared to EDLCs.^1–9^ Typically faradaic pseudocapacitors have further two different types comprising redox pseudocapacitance (surface or near-surface redox reactions) and intercalation pseudocapacitance (electrolyte ions intercalate into the tunnels/layers of the electrode materials accompanied by faradaic charge-transfer with no phase transitions).^10,11^

Transition metal oxides and hydroxides, transition metal dichalcogenides and carbon based materials have been studied as electrode materials for charge storage applications.^12^ After the discovery of graphene, rapid development of other new emerging layered structures such as MXenes,^13^ layered nanoclay, phosphorene, bismuthene and 2D graphene analogues have excellent charge storage capabilities.^14^ Many structural evolutions, chemical changes and hybridizations of previously used traditional materials in industry are being made to utilize the synergistic effect of constituent materials to ultimately achieve better electrochemical performance. These hybrids/nanocomposites integrate the advantageous attributes and compensate for the disadvantages associated with its single components. New advances include porous 2D and 3D graphene materials,^15^ transition metal oxide–hydroxide heterostructure (*e.g.* Co_3_O_4_/Co(OH)_2_ heterostructure *via* interfacial layer control^12^), spinel-type Co_3_O_4_ and its modification in spinel cobaltites MCo_2_O_4_ (M = Co, Mn, Zn).^16^ Recently Huan Pang *et al.* work suggests that MOFs (metal–organic frameworks) and its various derivatives such as multimetallic MOFs (*i.e.* bimetallic and trimetallic MOFs)^17^ and MIL-96-Al^18^ with controllable shapes and sizes also possess enhanced charge storage (sulfur storage).

For a material to be an efficient supercapacitor electrode, there should be some spaces inside structure to store charge *e.g.* MnO_2_ exist in many structural phases (*i.e.*, α, β, γ, λ and δ) each of which differs in its shape, size and dimensions of tunnels. So the specific capacitance (*C*_s_) of β-MnO_2_ [narrow (1 × 1) tunnels] is lower while δ-MnO_2_ (interlayer separation ∼ 7 Å) exhibits high *C*_s_ values being layered structure.^2,19–21^ N. Munichandraiah *et al.* synthesized nanostructured MnO_2_ samples with different crystal structures, and investigated as electrode materials for electrochemical capacitors in aqueous 0.1 M Na_2_SO_4_ solution. The *C*_s_ values are 240 F g^−1^ for α-MnO_2_ and 236 F g^−1^ for δ-MnO_2_. Alternatively, they are as low as 9 F g^−1^ for β-MnO_2_ and 21 F g^−1^ for λ-MnO_2_.^19^

Beside materials containing intrastructural spaces, porous structures are becoming suitable candidates for supercapacitors. Recently Vlad and Balducci showed higher normalized capacitance relative to their BET surface areas of Ni_3_(hexaiminotriphenylene)_2_ metal–organic framework (MOF) as compared to activated carbons, carbon nanotubes, zeolite-templated carbon, carbide-derived carbon and graphene.^22^

Porous materials can be categorized on the basis of pore size *i.e.* macroporous (>50 nm), mesoporous (2–50 nm) and microporous (≤2 nm).^2,23–25^

Recently porous silicon has been widely studied for variety of applications, most specifically as anode material in lithium-ion batteries.^26–28^ Previously established synthetic route of mesoporous silicon from silicon is physical etching which require long duration, various pre-steps and involves many harsh corrosive reagents.^29^ Three dimensionally structured silicon replicas could be produced from parent silica diatom by magnesiothermic reduction (*i.e.*, vaporized/liquefied magnesium at higher temperatures reduces different metal oxides). Currently, magnesiothermic reduction, being simple and lower temperature method to convert silica to nanostructured silicon is trending which usually results in porous silicon contaminated by unreacted silica.^30^

We have developed a scheme to synthesize the mesoporous silicon by magnesiothermic reduction of mesoporous silica. The focus of this research was to keep intact the porosity of mesoporous silica in resultant silicon which can be used in the energy areas like supercapacitors and hydrogen generation in the form of composites. Ultimately, we can avail the advantage of this new technique by using it to produce mesoporous silicon having good cycling stability. Our developed synthetic route comprises of just two steps. Firstly, mesoporous silica was synthesized from silica precursor *via* soft template method. Resultant mesoporous silica was converted into mesoporous silicon by magnesiothermic reduction.

Polyaniline (PANI) due to its easy synthesis, high controllable electrical conductivity (due to its conjugated structure having delocalised p-electrons along its backbone) and environmental stability is considerably attractive choice among several conducting polymers (CPs).^31,32^ It offers many advantages being pseudo-capacitive electrode material containing high theoretical specific capacitance, as short path lengths for ionic transport allow faster ionic diffusion within the polymer network so energy is delivered relatively at rapid rate.^33^ Its high surface area in contact with the electrolyte, allows comparatively fast or high charge/discharge rates. However, the shrinkage and swelling occurring due to doping–dedoping phenomena result in poor mechanical characteristics and low cycle life limit (*i.e.* poor cycling stability) when used as individual electrode material.^34,35^ The strategies used to overcome the issue with its cyclic life mainly include irradiation, sonication during fabrication or compositing it with fillers which increase volume of polymer, enhances porosity and provide room for its swelling.^33^ Compositing with other materials such as carbon based materials (carbon nanotubes and graphene), inorganic oxides (such as SnO_2_, MnO_2_, TiO_2_), sulphides and hydroxides and other metal compounds have been proved to enhance cyclic stability as well as maximize the capacitance of resultant composites.^6,15,33,36–42^

In order to alleviate the limitation associated with PANI-matrix for charge storage application, PANI was added inside synthesized mesoporous silicon (which contains mixed pores within mesoporosity range) to increase its charge storage, cyclic stability and specific capacitance. Besides this, PANI was also added in the mesoporous silica under same conditions and was compared with simple PANI and PANI with mesoporous silicon. This convenient and scalable synthesis procedure developed here indicates a significant potential towards cost-effective electrode materials for practical applications. Moreover the surface area of mesoporous silicon (348 m^2^g^−1^) attained by magnesiothermic reaction is amongst the highest published in literature so far.^30^

## Experimental

### Materials

All the chemical reagents of analytical grade were purchased from Sigma-Aldrich and were utilized without any further treatment.

### Synthesis of mesoporous silica (SBA)

2 mL of Triton X-100 (TX-100) was added in 80 mL of 1.3 molar aqueous solution of HCl in a beaker and stirred overnight at room temperature. 5.5 mL tetraethylorthosilicate (TEOS) was then added dropwise to TX-100 and acid solution under vigorous stirring to achieve clear solution. After it, the mixture was kept static at 35 °C overnight, followed by hydrothermal treatment for 24 h at 100 °C in a Teflon-lined autoclave. White solid product was collected by filtration process. Later it was washed with water then dried for 3 h at 60 °C and calcined for 6 h at 550 °C.^26^ The attained sample was designated as SBA.

### Magnesiothermic reduction of SBA to mesoporous silicon (mSi)

SBA powder placed in an alumina crucible was evenly covered with flakes of magnesium (Mg). The weight ratio of Mg : SBA was 0.8 : 1. The loaded crucible was kept in a tube furnace under N_2_ flow (80 sccm) for 3 h at 680 °C.

1 SiO_2_ + 2Mg → Si + 2MgO

**Fig. 1:**
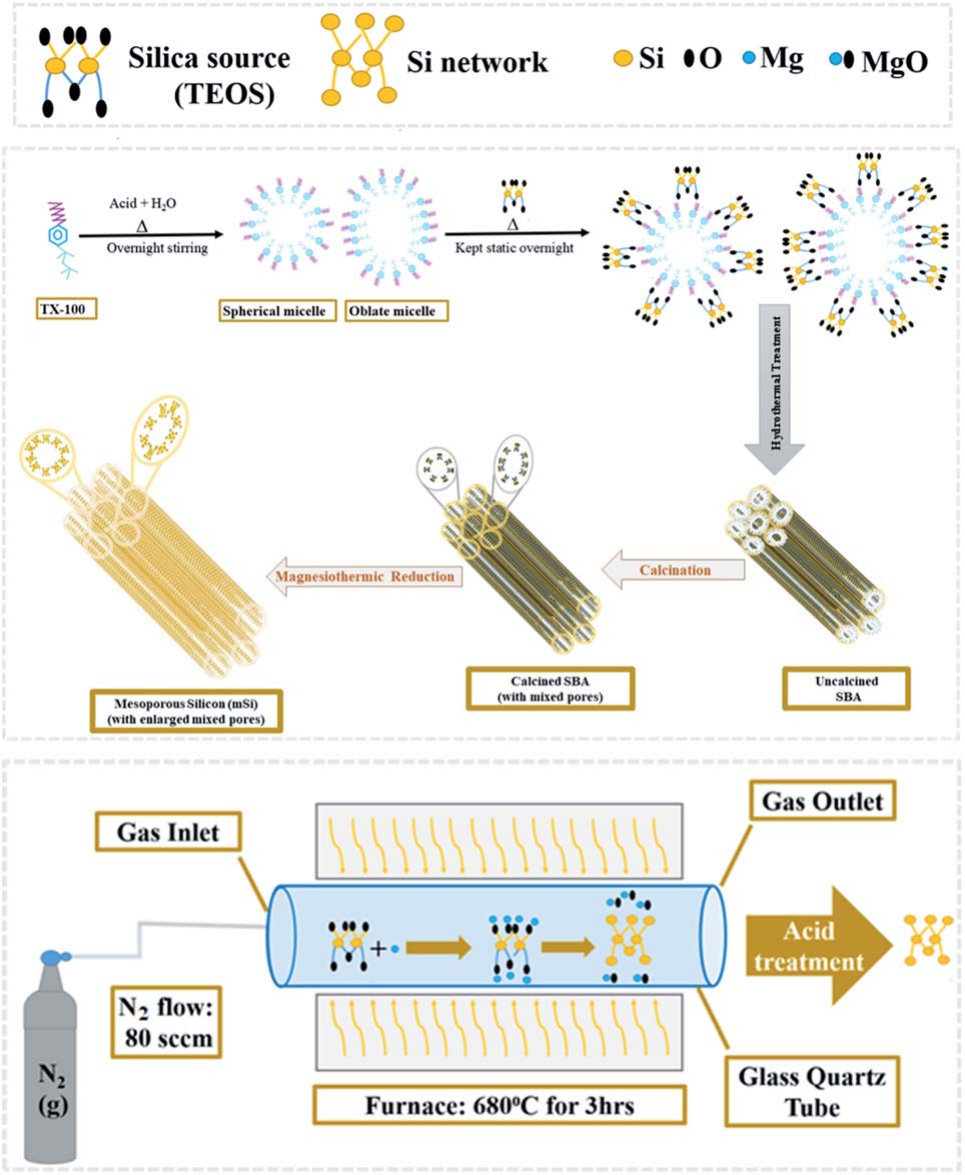
Schematic diagram of magnesiothermic reduction of mesoporous silica to mesoporous silicon.

When SiO_2_ (silica) and Mg (magnesium) react during magnesiothermic process, Si (mesoporous silicon) forms and MgO (magnesium oxide) is produced as a by-product (Fig. 1).

The powder thus obtained was washed with 1 M hydrochloric acid (HCl) and then vacuum filtered. A greyish brown fine powder of mesoporous silicon (mSi) was attained as end product.

### Synthesis of polymer (P) and polymer composites (mSi–P & SBA–P)

Aniline monomer was first dissolved into a dilute HCl aqueous solution. Aniline/aqueous acid solution was placed in an ice-salt bath to maintain the temperature at 4–5 °C. For synthesis of mesoporous silicon–polyaniline (mSi–P) and mesoporous silica–polyaniline (SBA–P) composites, the fillers (mSi and SBA respectively) were added in aniline/acid solution mixture at this stage. The polymerization of aniline in aqueous medium was done in the presence of an initiator *i.e.* ammonium persulfate, (NH_4_)_2_SO_4_. Aqueous solution of (NH_4_)_2_SO_4_ in ice-cold water was slowly added to the prepared mixture under moderate stirring condition to get homogeneous mixture. Then stirring was stopped and the whole homogeneous mixture was placed to age at 4–5 °C for 16 h under static condition. Resultant polymer/composite was filtered and washed with distilled water. Same procedure was followed for polyaniline (P) without addition of mSi or SBA.

## Characterization

XRD and SAXS patterns of the samples were collected using a Bruker D8 Advance diffractometer. SAXS data was collected by keeping the X-ray source fixed at 0.1 degree. XPS data was collected using a Scienta Omicron nanotechnology GmBH equipped with a hemispherical analyser. Nitrogen sorption isotherms were obtained using an ASAP 2020 system (Micromeritics) operated at 77 K. The samples were degassed at 200 °C for 4 h prior to the analysis. The Brunauer–Emmett–Teller (BET) method based on the adsorption data (within the range of *P*/*P*_0_ = 0.05–0.20) was used to calculate the specific surface area. The total pore volume was obtained from the adsorbed amount of N_2_ at *P*/*P*_0_ = 0.99. Barrett–Joyner–Halenda (BJH) analysis was used on the adsorption branch to evaluate the average pore diameter. Hitachi Su-70 Schottky field emission microscope was used for SEM. TEM analysis was done using a FEI TECNAI G2 microscope operated at 200 kV. The samples were prepared by dispersing the materials in acetone and depositing them on hollow carbon grids. FT-IR data was collected on a Bio-Rad Excalibur FT-IR Spectrometer in the range 400–4000 cm^−1^ using pellets of KBr at ambient temperature.

## Electrochemical measurements

The electrochemical properties of the synthesized samples were studied in the three-electrode system with 1 M H_2_SO_4_ aqueous solution as electrolyte. Reference electrode was saturated calomel electrode (SCE) while graphite rod was utilized as counter electrode. The working electrodes were prepared by mixing active materials and polytetrafluoroethylene (PTFE) as the binder. A Gamry REF3000-electrochemical workstation was used to collect the cyclic voltammetry (CV), electrochemical impedance spectroscopy (EIS) and galvanostatic charge/discharge (GCCD) data. CV scans were carried out in the potential range from −0.2 V to 1 V at various scan rates. Cyclic stability was checked for 50 cycles at 20 mV s^−1^. The specific capacitance can be determined by integration of the CV curve to calculate the average area under the curve for a complete cycle using the relation^43^ given in the eqn (2).2
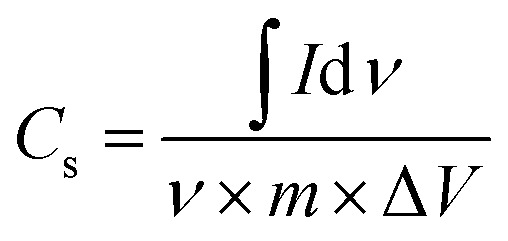
where ∫*I*d*v* = total integrated area of curve, Δ*V* = potential window, *ν* = scan rate, *m* = mass of active material.

Specific capacitance of three electrode system can be estimated also by slope of discharge curve of GCCD measurement by following formula^44^ given in eqn (3).3
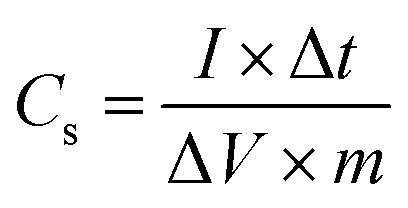
where *I* = discharge current, *m* = mass of active material, Δ*V*/Δ*t* = slope of discharge curve.

## Results and discussion

### Structural studies

#### X-ray diffraction (XRD) analysis

**Fig. 2:**
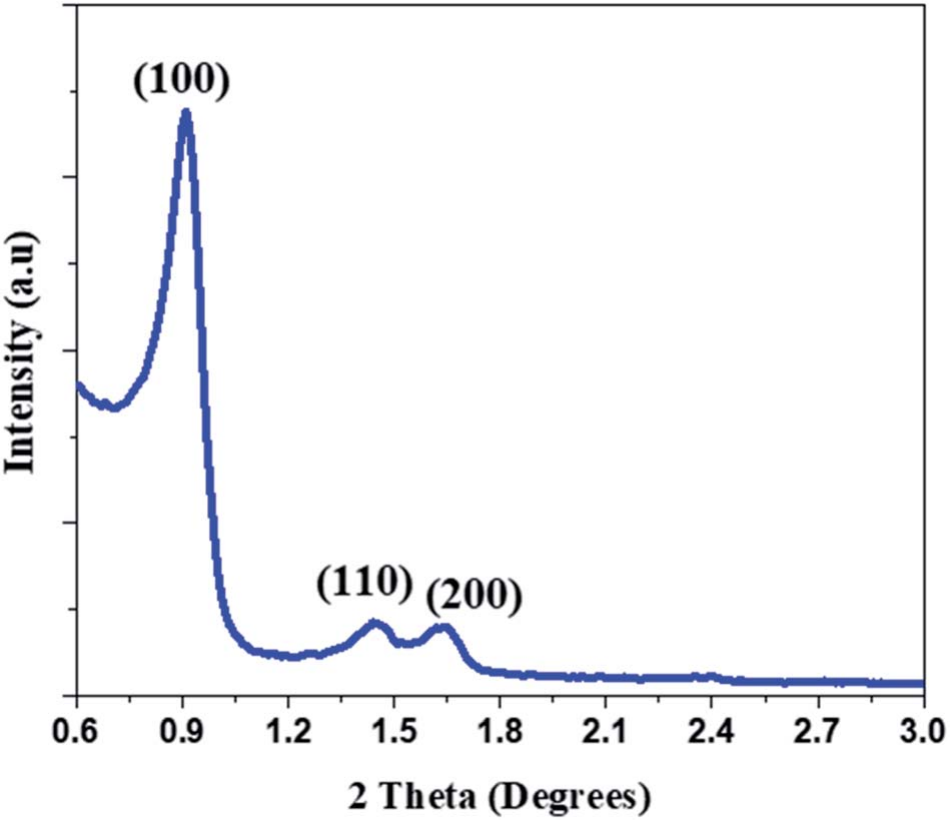
Small angle X-ray diffractogram of the as synthesized mesoporous silica (SBA).

The small angle XRD pattern of mesoporous silica (SBA) sample shows (Fig. 2) a strong (100) reflection arising from the edge to edge diffraction from the pores which is characteristic of the hexagonal pore ordering in SBA (ICCD#: 00-047-0718). The 200 reflection corresponds to a pore size below 10 nm.

**Fig. 3:**
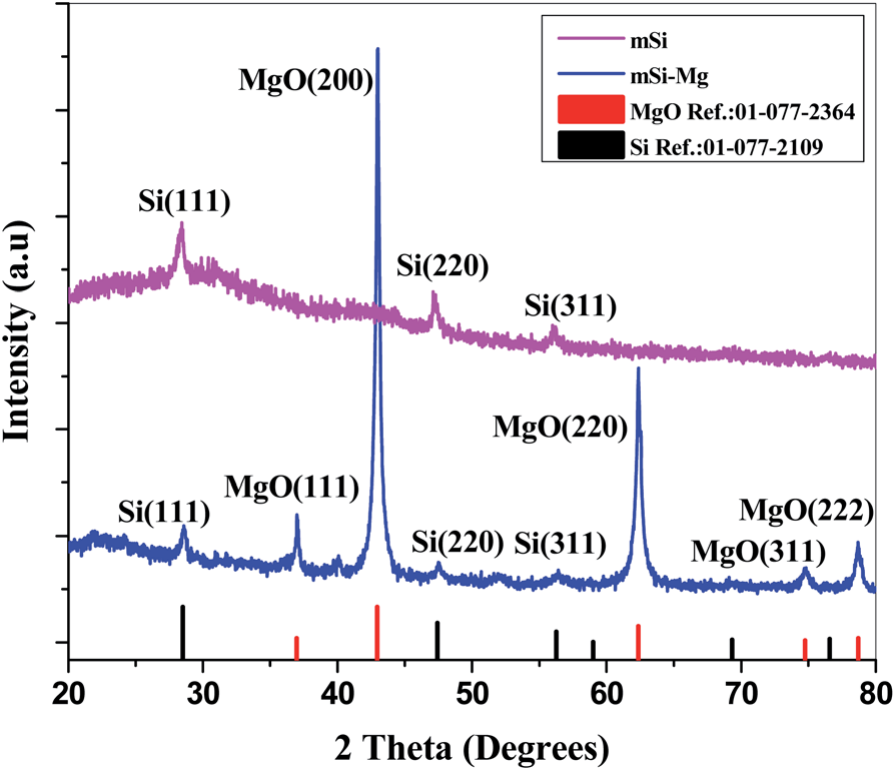
X-ray diffractograms of mesoporous silicon reduced from mesoporous silica before (mSi–Mg) and after (mSi) the acid treatment.

In the XRD patterns of as reduced mSi, peaks from MgO were observed (ICCD#: 01-077-2364, 01-077-2109) (Fig. 3) along with reflections from Si indicating the complete reduction of SBA to mSi. After acid washing, the reflections from MgO disappeared and only the three prominent peaks of Si at 28.4°, 47.3° and 56.1° were present (ICDD#: 00-026-14181). There is no evident peak of mesoporous silica so it shows that magnesiothermic reduction with consequent acid washing left no residues of parent material (Fig. 3).

PANI exhibits X-ray diffraction peaks at 14.4°, 19.6°, and 25.4° for (121), (113) and (322) reflections respectively.^45,46^ Intensity of these peaks decreases slightly in XRD diffractograms of composites of mSi–P & SBA–P, which is due to the interaction between PANI and filler (ESI Fig. S1†).

### Morphological studies

#### Transmission electron microscopy (TEM)

**Fig. 4:**
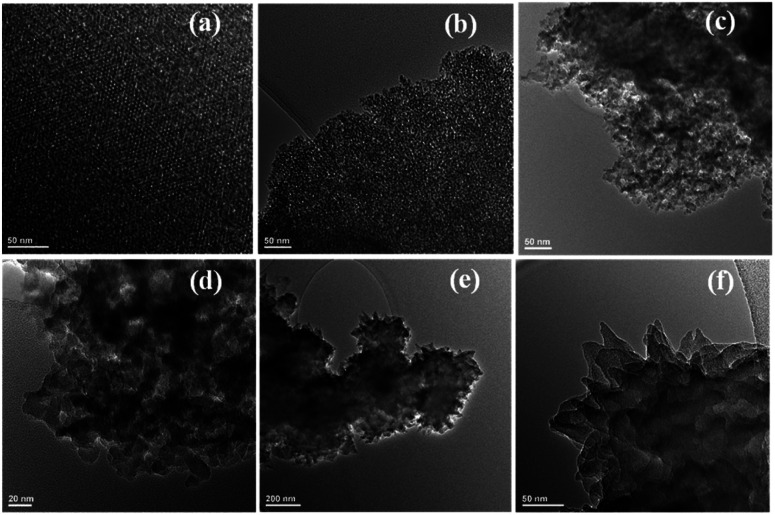
Transmission electron micrographs of (a and b) SBA depicted at two different locations (scale bar 20 nm), (c) mSi (scale bar 50 nm) (d) mSi (scale bar 20 nm), (e and f) mSi–P (scale bars 200 nm and 50 nm).

**Fig. 5:**
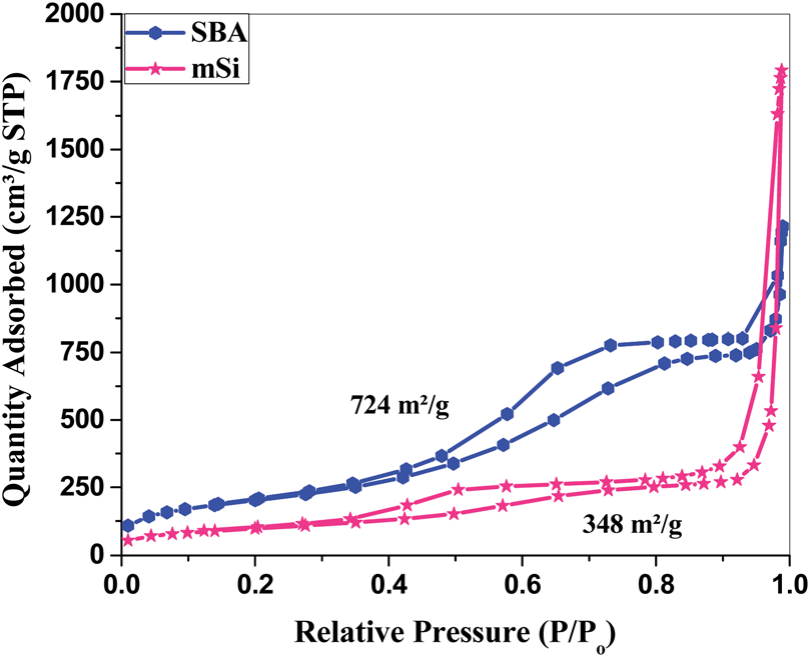
Surface area analysis – Brunauer–Emmett–Teller (BET): N_2_ adsorption–desorption isotherm of SBA and mSi.

TEM micrographs of SBA (Fig. 4(a) and (b)) are representative of mesoporous silica prepared with TX-100. The micrographs show that the mesoporous silica has a three-dimensional hexagonal pore structure. Micrographs of mSi (Fig. 4(c) and (d)) show that the porosity of the parent SBA are retained after magnesiothermic reduction. The pores are enlarged upon formation of Si crystals. TEM micrographs of SBA and mSi show the mixed pore structures which is also evident by nitrogen sorption isotherms (Fig. 5). It should be noted that the mSi (Fig. 4(c) and (d)) is very sensitive to the electron beam, and that the pore structure collapses upon exposure to the electron beam. TEM micrographs of mSi–P (Fig. 4(e) and (f)) show polyaniline is protruding out of the mesoporous silicon at the edges.

### Surface studies

#### Nitrogen physisorption

**Fig. 6:**
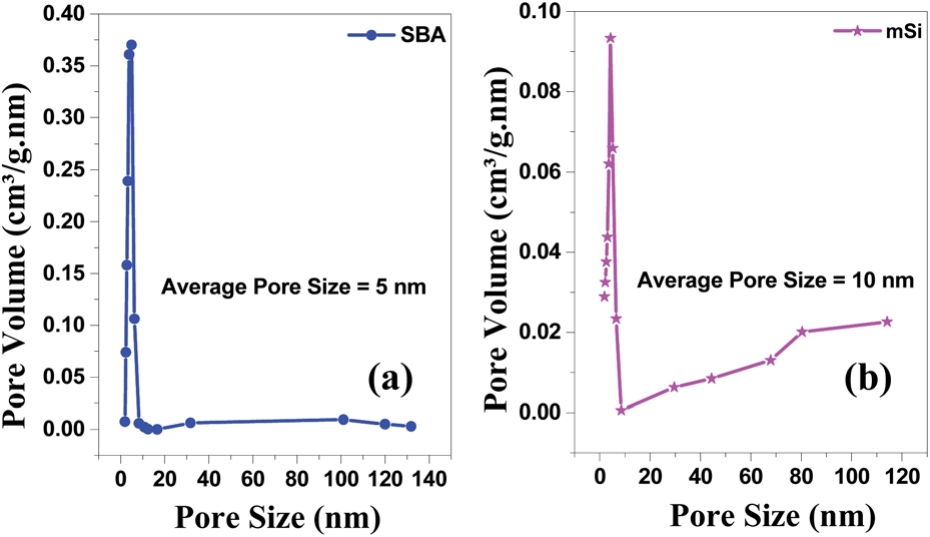
BJH pore size & volume analysis – Brunauer–Emmett–Teller (BET) of (a) SBA and (b) mSi by Halsey: Faas correction.

The N_2_ adsorption–desorption isotherm for SBA (Fig. 5) is of type IV with hysteresis loop at high pressure which is typical characteristic of mesoporous materials. The hysteresis loop at *P*/*P*_0_ = 0.4–0.8 is of type H1, typical for cylindrical pores, while at higher pressure a type H3 hysteresis associated with slit-shaped pores is observed.^47–52^ The mixture of pores can be due to the reason that the surfactant TX-100 can form both spherical and oblate micelles depending upon the arrangement of oxyethylene chains inside the micelles.^53–55^ The pores of SBA are of the size ∼5 nm, and the material shows a narrow pore size distribution, see Fig. 6. The specific surface area of SBA is 724 m^2^ g^−1^.

For mSi, the N_2_ adsorption–desorption isotherm still shows a type H1 hysteresis loop followed by a type H3 hysteresis. This indicates that the metallic Si retains part of the mesopores, even though some pore shrinkage is observed in the pore size distribution in Fig. 6. The strong H3 loop is most probably due to the slit shaped pores formed between Si crystals, see Fig. 4.^47–52^ The specific surface area of mSi is 348 m^2^ g^−1^. This significant decrease in specific area is due to a decreased mesoporosity upon the formation of crystalline Si. The narrow pore size distribution of mSi indicates a homogeneous material reduction.

#### X-ray photoelectron spectroscopy (XPS)

**Fig. 7:**
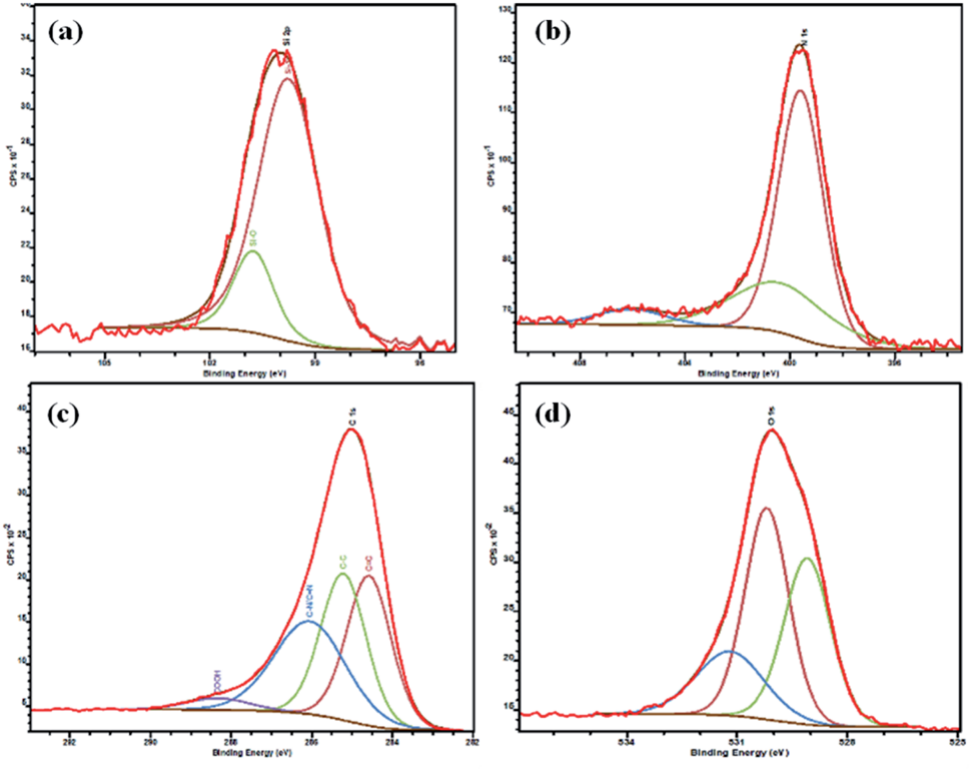
X-ray photoelectron spectroscopy (XPS) of mSi–P showing peaks of (a) Si 2p, (b) N 1s, (c) C 1s and (d) O 1s.

The chemical bonding status of mSi was elucidated by using XPS to further verify the complete magnesiothermic reduction reaction (Fig. 7). The Si 2p spectrum of the mSi contains a peak at 99.5 eV for Si–Si (elemental silicon) bonds. Upon deconvolution of this peak depicts that a low intensity peak of Si–O exists which is due to the natural oxidation of surface of mSi particles in airy and humid environment at room temperature and not from the residual SBA.

In the XPS survey spectrum of mSi–P, peaks from Si 2p were due to mSi while peaks from C 1s and N 1s were observed due to the presence of polyaniline in the composite. N 1s was fitted into three components arising from the –NH_2_^+^, =NH^+^, and =N– moieties in PANI. C 1s was fitted into four components consisting of C–C, C=C, C–N/C=N, and COOH groups. The three components under the O 1s spectra corresponds to adsorbed O, Si–O and, C–O groups.

### Electrochemical studies

Cyclic voltammetry (CV) measurements were performed in 1 M H_2_SO_4_ aqueous solution as the electrolyte to investigate the electrochemical behaviour of samples. The CV scans were taken at scan rates of 5 to 250 mV s^−1^ (*i.e.*, 5, 10, 20, 50, 100, 150, 200, 250 mV s^−1^) in voltage range of −0.2 to 1 V (*versus* SCE) for all samples.

**Fig. 8:**
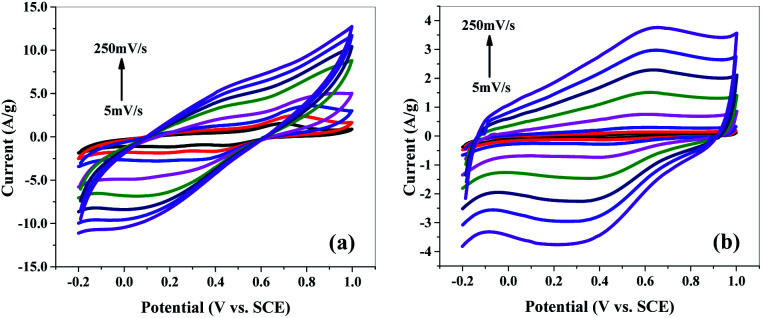
Cyclic voltammogram (CV) of (a) mSi–P and (b) mSi at different scan rates on GCE in 1 M H_2_SO_4_ electrolyte *vs.* SCE.

Cyclic voltammograms of polyaniline (P) (Fig. S4(a)†) at various scan rates exhibit a redox peak which is attributed to the transition of PANI from leucoemeraldine (semiconducting state) to emeraldine (conductive form). This redox process caused pseudo capacitance of PANI. Apparently, the mSiP composite (Fig. 8(a)) has a similar electrochemical response as that of the P, but peak current of mSi–P increases greatly which implies a larger electrode capacitance. High electrochemical utilization of mSi–P is due to its larger specific surface area as compared to P and hence more electroactive sites.

The PANI matrix is electrochemically permeable *i.e.* it does not block the counter-ions to reach the mSi because of the close contact established between the two during *in situ* polymerization inside the pores of mSi.

Comparison of the shapes of the cyclic voltammograms (Fig. 8(a), S4(b) and (c)†) depicts that the contribution of the mSi and SBA to the total capacitance of mSi–P and SBA–P composite electrodes is of the double-layer type which is also apparent in the form of CV curves for pure mSi (Fig. 8(b)) and SBA (Fig. S4(b)†). The electrochemical behaviour of mSi–P (Fig. 8(a)) is still better than SBA–P (Fig. S4(c)†) which is because of enlarged pores of mSi than SBA which facilitate the charge transfer. P (Fig. S4(a)†) is showing good capacitance response but its efficiency decreases after multiple cyclic charge–discharge phenomena due to changes in its volume and physical properties which is inevitable in its pure polymer matrix.

The rectangular shape and mirror images are observed in the CV curves for all samples (Fig. 8 and S4†), indicating high electrochemical reversibility.^36–38,56,57^

At 5 mV s^−1^ scan rate and 1.2 V potential, the specific capacitance of mSi is 19.85 F g^−1^ while for P its value is 178 F g^−1^. Specific capacitance of mSi–P is 214.45 F g^−1^.

**Fig. 9:**
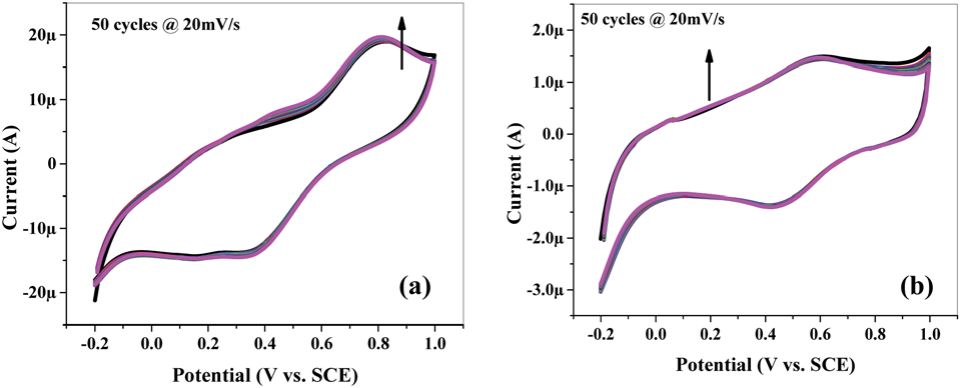
Cyclic voltammetry (CV): cyclic stability of (a) mSi–P and (b) mSi at scan rate of 20 mV s^−1^ for 50 cycles on GCE in 1 M H_2_SO_4_ electrolyte *vs.* SCE.

**Fig. 10:**
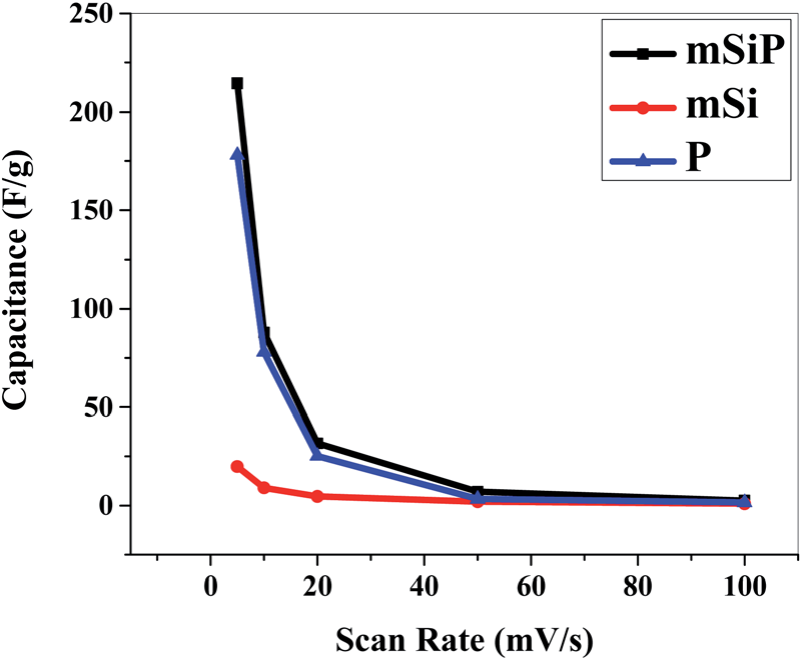
Scan rate *vs.* specific capacitance of PANI, mSi and mSiP.

Fig. 9 and S5† show the cyclic voltammograms recorded at 20 mV s^−1^ scan rate for 50 cycles in 1 M H_2_SO_4_ aqueous electrolyte solution. The observed CV scans are quite stable for 50 cycles and no significant decrease in current is observed for any material which depicts that materials have high cycling stability. Fig. 9(a) is showing the good cyclic stability of the electrode which is due to the cohesive inter-molecular contact between P and mSi which inhibits the dissolution of filler. Fig. 10 and S6† show specific capacitance decreases by increasing scan rate.

Further, relative contributions of the capacitance from the bulk or surface mechanism is derived by analyzing the CV data at various scan rates (5–250 mV s^−1^) shown in Fig. S10† and using the power law^58,59^ given in eqn (4).

4 *i* = *av*^*b*^

According to power law, slope of the plot log(*i*) *i.e.* redox peak current *versus* log(*v*) *i.e.* various scan rates provides the *b*-value which can be used to estimate the controlling mechanism of the active material in electrode. For ideal capacitor type materials, *b*-value is close to 1 where pseudocapacitive behavior is dominant. If the value of *b* is close to 0.5, it depicts the material is battery type and contains diffusion controlled phenomenon as dominant one. Fig. S10† shows a plot between log(*i*) and log(*v*) showing the cathodic and anodic peak currents of mSi–P as indicated in Fig. 8(a). The *b*-values from the anodic and cathodic peaks are 1.009 and 1.022 respectively. It suggests that total contribution to capacitance is mainly due to peudocapacitance phenomenon and mSi–P is showing mainly capacitive behavior. Using the Dunn method of differentiation,^60^ it is estimated that 99.78% of the total capacitance is from pseudocapacitive contribution and 0.22% is diffusion controlled behavior.

EIS is performed in the frequency range of 10^6^ to 10^−1^ Hz (at AC voltage = 10 mV rms, open circuit potential amplitude = 0.34 and stabilization time = 10 s) to evaluate the resistance of electrochemical phenomenon.

**Fig. 11:**
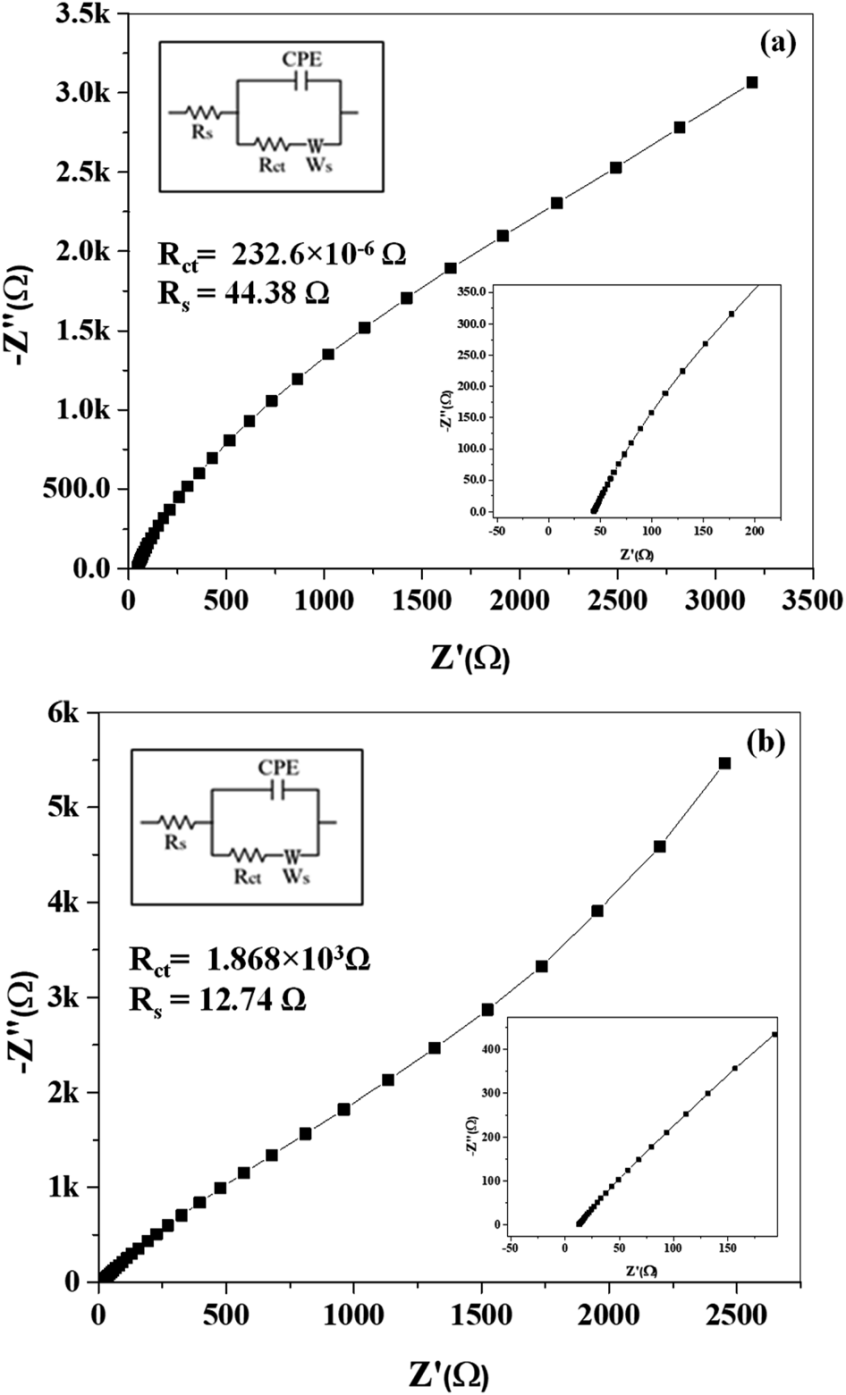
EIS (electrochemical impedance spectroscopy): Nyquist plots of (a) mSi–P and (b) mSi *vs.* OC (open circuit) at 10 mV rms AC perturbation (in 1 M H_2_SO_4_ electrolyte) with equivalent circuit diagram.

Fig. 11 and S7† show the Nyquist plots of samples. Fig. 11(a) shows the frequency response of mSi–P/1 M H_2_SO_4_ (aq.) system in the plotted form of two impedance components against each other, one of which is real component *i.e. Z*′ and other one is imaginary component *i.e. Z*′′. The Nyquist plots are fitted using an equivalent circuit model shown in the inset of Fig. 11, where *R*_s_ is solution ohmic resistance, *R*_ct_ is charge transfer resistance, CPE is constant phase element and *W* is Warburg impedance. In Fig. 11(a), absence of semicircle in the domain of high frequency shows less *R*_ct_. *R*_ct_ calculated by fitting equivalent circuit model is 232.6 × 10^−6^ Ω. *R*_ct_ existing between mSi–P electrode and 1 M H_2_SO_4_ (aq.) electrolyte is considerably less due to the highly conductive cross-linked mSi and PANI. It may relate to a high material electrochemical activity (pseudo capacitance), and indicates the surface properties of mSi–P electrode is favouring facile fast charge transfer kinetics within electrodes.^61^

Large impedance values observed for mSi electrodes in Fig. 11(b) are indicative of high *R*_ct_ (1.868 × 10^3^ Ω) which is greater than that of mSi–P composite (Fig. 11(a)).

In Fig. 11(a) at low frequency region a steeper curve is evident of decreased Warburg impedance which depicts the accelerated diffusion and adsorption rate of counter-ions of electrolyte in the system (in/on the electrode material). The reason behind it is that the network of PANI enwrapping mSi (having mesoporous configuration) with high surface area provide more space with short and equal diffusion path length for transportation of redox species (*i.e.* counter-ions) of 1 M H_2_SO_4_ electrolyte consequences in excellent capacitive behavior.

**Fig. 12:**
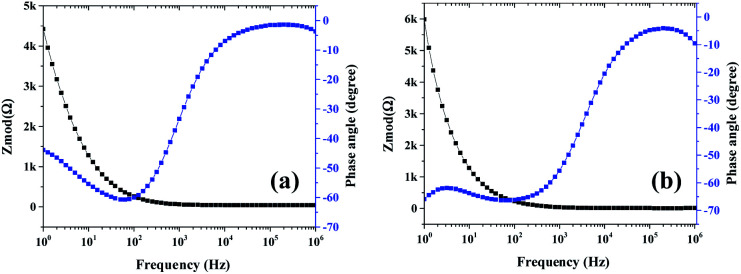
EIS (electrochemical impedance spectroscopy): Bode plots of (a) mSi–P and (b) mSi *vs.* OC (open circuit) at 10 mV rms AC perturbation (in 1 M H_2_SO_4_ electrolyte).

Nyquist plot (Fig. 11(b)) for mSi shows larger *R*_ct_ and Warburg impedance as compared to mSi–P; Fig. 11(a) which is due to high obstruction in the movement of counter-ions and higher variations in ion diffusion path lengths respectively.

The slope of each electrode (Fig. 11 and S7†) decreases with increasing AC frequency (potential) which demonstrates an enhanced Warburg resistance which is consequently causing reduction in effective charge storage at the electrode. Fig. 12 and S8† show the Bode plots of samples.

**Fig. 13:**
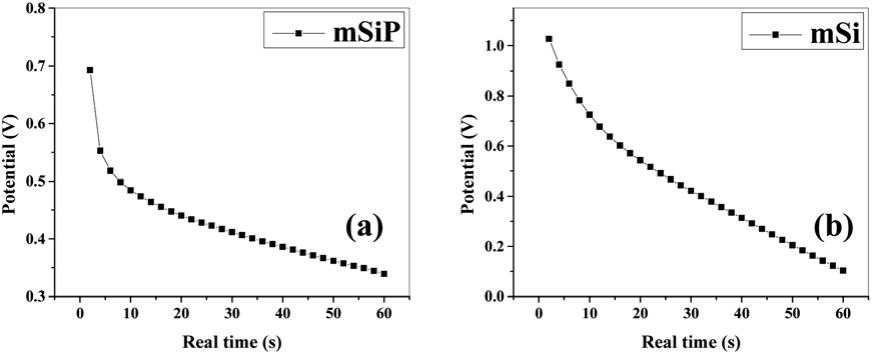
GCCD (galvanostatic cyclic charge–discharge): discharge curve; potential *vs.* real time (a) mSi–P (at charge density = 0.187 A g^−1^) and (b) mSi (at charge density = 0.210 A g^−1^) in 1 M H_2_SO_4_ electrolyte.

The electrochemical stability was examined by galvanostatic charge/discharge (GCCD) measurements in 1 M H_2_SO_4_ aqueous solution by consecutive charge–discharge cycles within the potential range of −0.2 to 1 V at discharge current of 1 μA and capacity of 0.0025 A h. GCCD measurements are executed to attain the quantitative information of the electrochemical capacitance for mSi–P, mSi, P, SBA and SBA–P electrodes (Fig. 13 and S9†).

The higher capacitance observed for mSi–P composite is due to low *R*_ct_ (also discussed in impedance analysis) which is the consequence of its unique morphology (with more electroactive sites due to mesoporosity of mSi for reversible redox reaction) while having continuous conductive network of PANI.

The specific capacitance calculated by discharge curve of GCCD curves for mSi–P is equal to 68.21 F g^−1^ at 0.187 A g^−1^ current density.

**Fig. 14:**
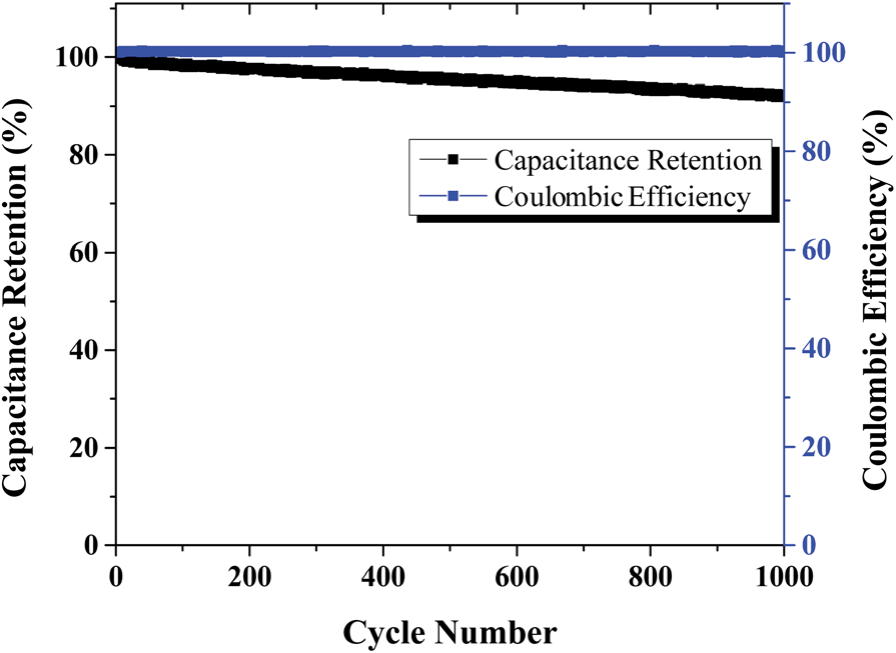
GCCD (galvanostatic cyclic charge–discharge): capacitance retention and coulombic efficiency of mSi–P *vs.* cycle number for 1000 cycles (in 1 M H_2_SO_4_ electrolyte).

Capacitance retention and coulombic efficiency of mSi–P for 1000 cycles in 1 M H_2_SO_4_ electrolyte is shown in Fig. 14.

## Conclusions

We have reported a facile and cost effective method to obtain mesoporous silicon with a remarkably high surface area (348 m^2^ g^−1^) by magnesiothermic reduction of mesoporous silicon (SBA). The conducting mesoporous network can be used to host small polymeric molecules such as PANI for most of applications. As supercapacitor electrode, mSi–PANI composite was able to achieve specific capacitance of 214.45 F g^−1^ at 5 mV s^−1^. The composite demonstrates improved charge storage performance and excellent cyclic stability (100% capacitance retention and 91.87% coulombic efficiency at 0.18 A g^−1^ after 1000 cycles). The results demonstrate that the pseudocapacitive contribution in total specific capacitance is dominant. The predominant pseudocapacitive behaviour is attributed to synergistic effect between mesoporous silicon and polymer matrix. This work suggests a simple and scalable route for the development of mesoporous silicon based hybrid composites favourable to supercapacitor applications.

## Author contributions

MS: supervision, reviewing, and editing, YK: conceptualization, supervision, administration, resources, reviewing, and editing, EB: help in synthesis of mesoporous silica and TEM analysis, AR: BET measurement and analysis, SA: SEM analysis, SK: methodology, investigation, formal analysis, SN: methodology, investigation, formal analysis, writing-original draft preparation, validation, data curation.

## Conflicts of interest

There are no conflicts to declare.

## Supplementary Material

RA-012-D2RA01829B-s001
