# Supplementary material for: Polyaniline inside the pores of high surface area mesoporous silicon as composite electrode material for supercapacitors
Source: RSC Adv. 2022 Jun 10;12(27):17228–36. doi: 10.1039/d2ra01829b (PMC9185315; doi:10.1039/d2ra01829b)
Supplement: RA-012-D2RA01829B-s001 [file RA-012-D2RA01829B-s001.pdf]

Electronic Supplementary Material (ESI) for RSC Advances.
This journal is © The Royal Society of Chemistry 2022

Polyaniline Inside the Pores of High Surface Area Mesoporous Silicon as Electrode Material for Supercapacitors

Saima Nawaz[a,b], Yaqoob Khan[b*] Shaimaa A.M Abdelmohsen[c], Sadia Khalid[b], Emma M. Björk[d], Asim Rashid[e], and M. Siddiq[a*]

[a.] *Department of Chemistry, Quaid-i-Azam University, Islamabad 45320, Pakistan.*
[b.] *Nanoscience and Technology Department, National Centre for Physics, QAU Campus, Shahdra Valley Road, Islamabad 45320, Islamabad, Pakistan.*
[c.] *Department of physics, college of science, Princess Nourah bint Abdulrahman University. P.O.Box 84428, Riyadh 11681, Saudi Arabia*
[d.] *Nanostructured Materials, Department of Physics, Chemistry and Biology (IFM), Linköping University, SE-581 83 Linköping, Sweden.*
[e.] *Department of Physics, Pakistan Institute of Engineering and Applied Sciences, Islamabad 45650, Pakistan.*
**Address correspondence to :*
Email address:m_sidiq12@yahoo.com
Tel.: +92 5190642147
Email:yaqoob@ncp.edu.pk (YK),
Tel: +923455235423
Fax: +92512077389

X-ray Diffraction (XRD) Analysis

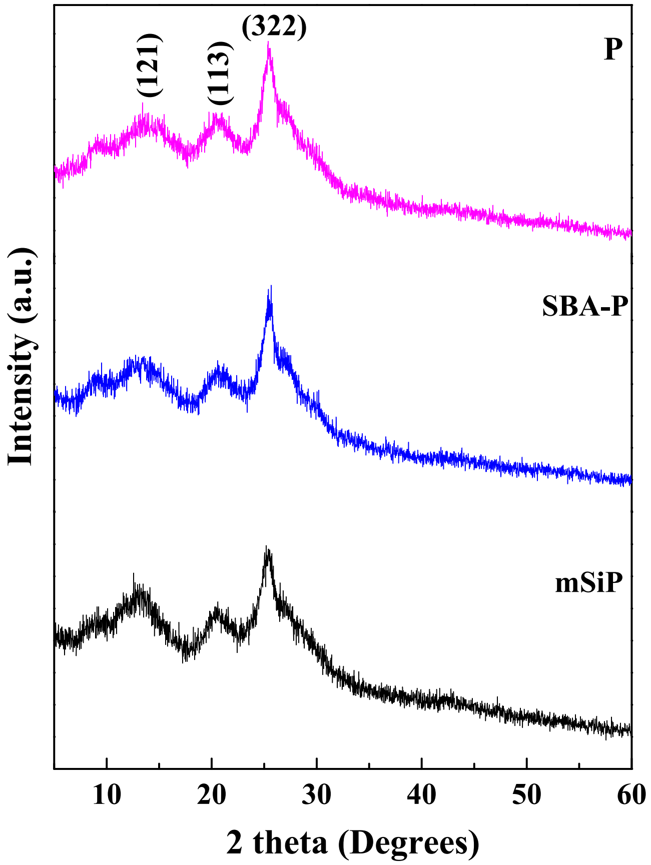

Figure S1: X-ray diffraction (XRD) pattern of polyaniline (P), SBA-P and mSi-P composites.

Scanning Electron Microscopy (SEM)

SEM micrographs of powdered sample of mSi (mesoporous silicon) shows the agglomerated nanoparticles, while the micrograph of pure polyaniline (P) is showing big chunks i.e. aniline monomers polymerized in amorphous structures. The SEM image of mSi-P (mesoporous silicon/PANI composite) showed enwrapped silicon nanoparticles by polyaniline, where the change in overall texture of polymer is clearly depicted.

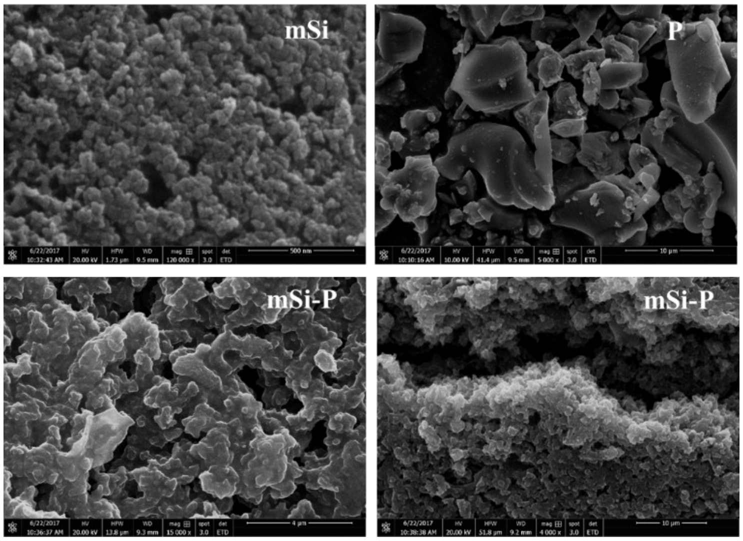

Figure S2 (a): Scanning Electron Microscopy (SEM) images of mSi, P and mSi-P composite.

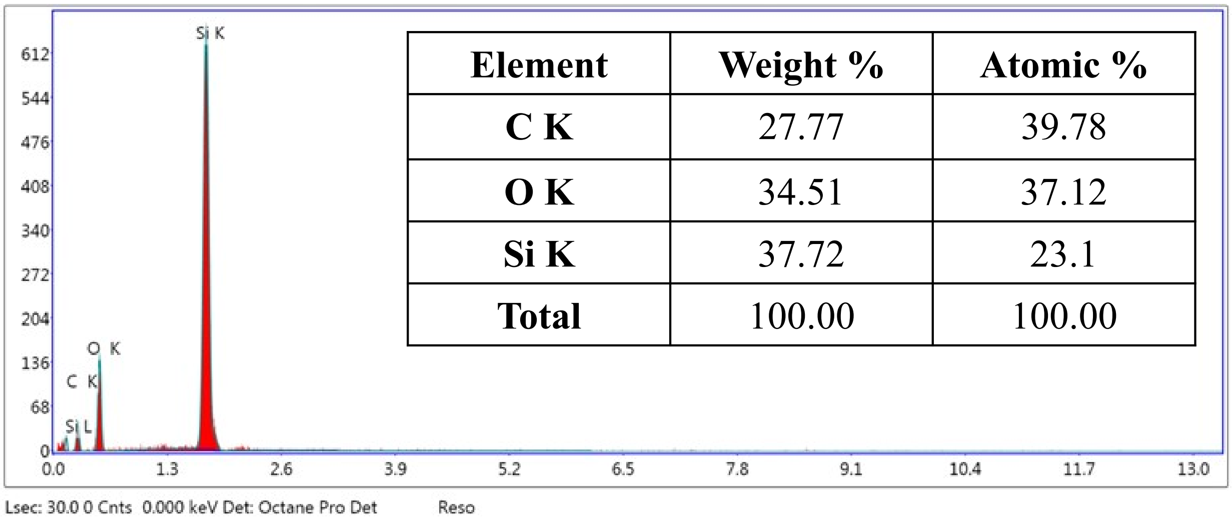

Element	Weight %	Atomic %
C K	27.77	39.78
O K	34.51	37.12
Si K	37.72	23.1
Total	100.00	100.00

Figure S2 (b): Energy dispersive X-ray (EDX) pattern of mSi-P composite. (The sample was mounted on carbon tape.)

Fourier transform infrared spectroscopy (FT-IR)

The characteristic IR peaks of SBA can be attributed to the stretching vibration peak of Si-O-Si bands & O–H bonds in silanol groups and adsorbed water molecules, which can be also observed in the PANI/SBA (SBA-P) composite. PANI exhibited its vibrations for N-H stretching, C=N stretching of secondary aromatic amine, C=C asymmetric stretching of quinnoid, C=C stretching benzenoid), C-C bending vibration, C-N stretching of secondary aromatic amine, C-H in-plane deformation in 1,4-disubstituted benzene and C-H out of plane deformation. The characteristic peaks of SBA-P composite emerge in both SBA and PANI, demonstrating the existence of both components in the composites. PANI/mSi composite (mSi-P) also show vibrational signature peaks of PANI.

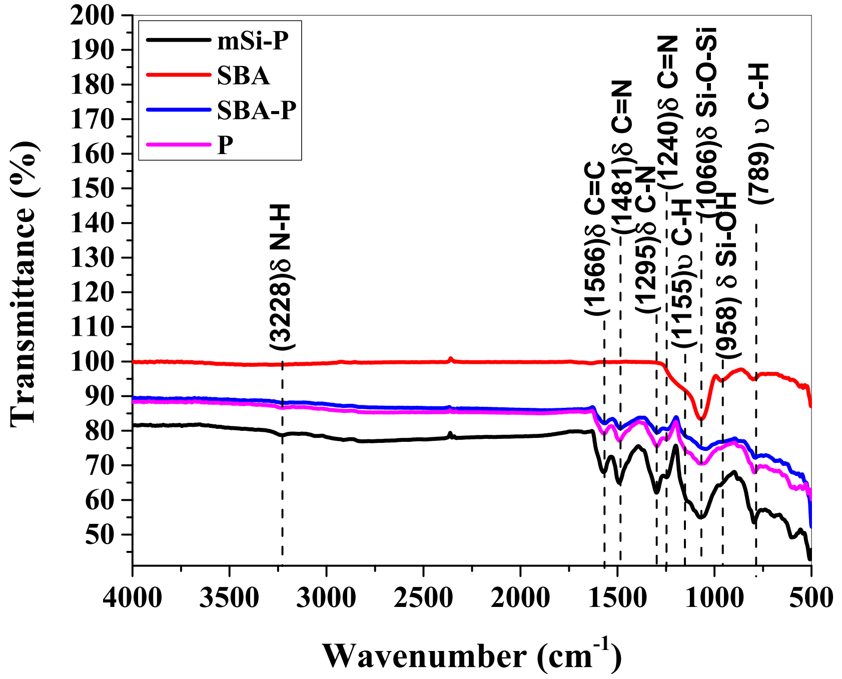

Figure S3: Fourier transform infrared spectroscopy (FT-IR) of SBA (mesoporous silica), polyaniline (P) and composites (mSi-P and SBA-P).

Cyclic voltammogram (CV) in 1M H_2SO_4 electrolyte vs. SCE.

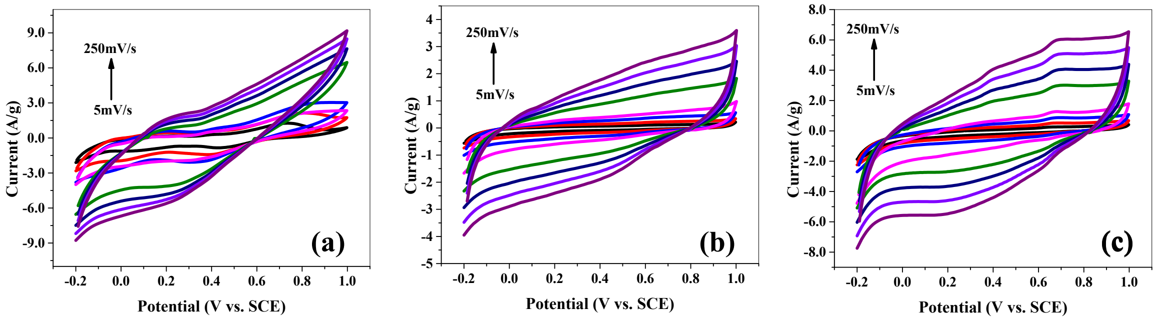

Figure S4: Cyclic voltammogram (CV) of (a) P, (b) SBA and (c) SBA-P at different scan rates on GCE in 1M H_2SO_4 electrolyte vs. SCE.

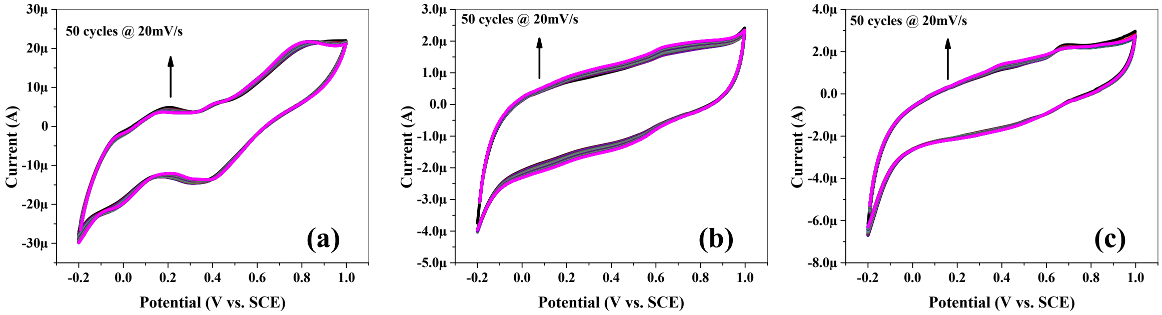

Figure S5: Cyclic voltammetry (CV): cyclic stability of (a) P, (b) SBA and (c) SBA-P at scan rate of 20mV/s for 50 cycles on GCE in 1M H_2SO_4 electrolyte vs. SCE.

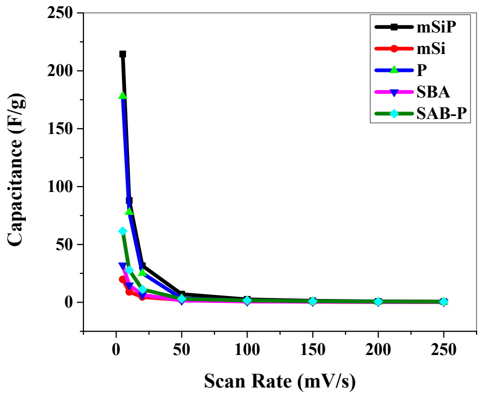

Figure S6: Scan rate vs. Specific capacitance.

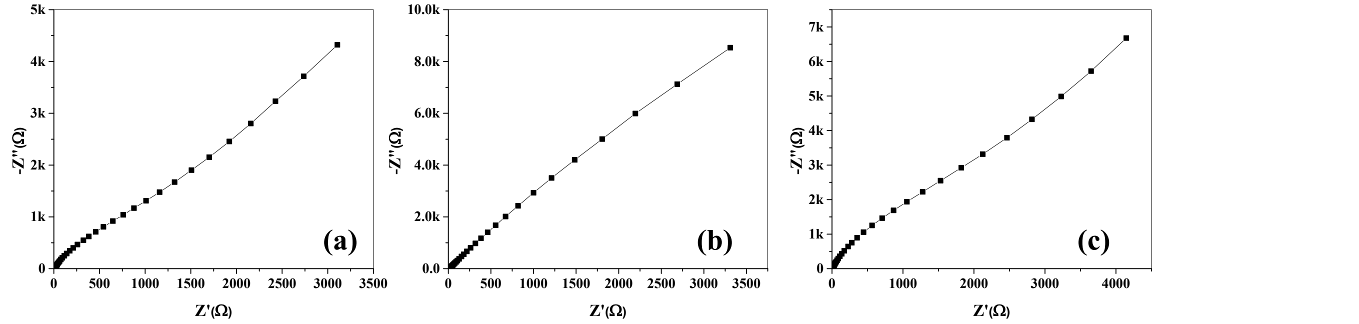

Figure S7: EIS (Electrochemical Impedance Spectroscopy): Nyquist plots of (a) P, (b) SBA and (c) SBA-P vs. OC (open circuit) at 10mv rms AC perturbation (in 1M H_2SO_4 electrolyte.

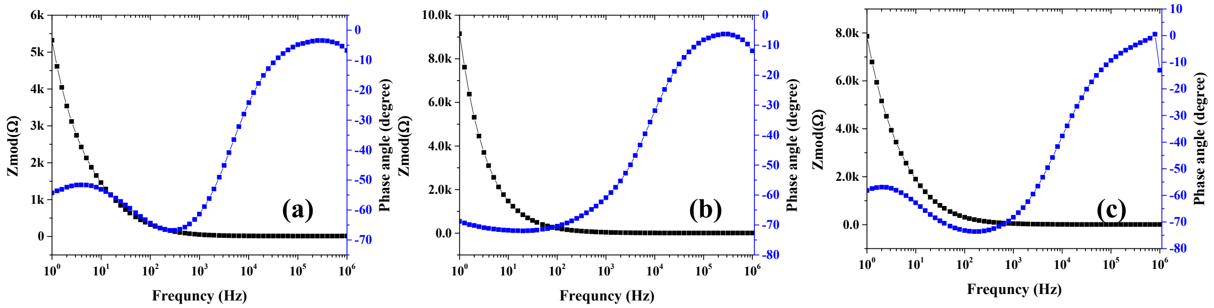

Figure S8: EIS (Electrochemical Impedance Spectroscopy): Bode plots of (a) P, (b) SBA and (c) SBA-P vs. OC (open circuit) at 10mv rms AC perturbation (in 1M H_2SO_4 electrolyte.

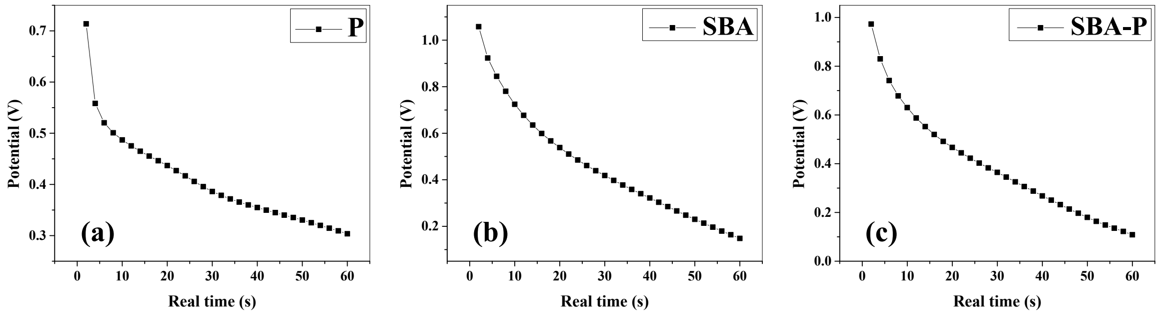

Figure S9: Galvanostatic cyclic charge discharge (GCCD): Discharge Curve; Potential vs. real time (a) P (at charge density= 0.143 A/g), (b) SBA (at charge density= 0.250 A/g) and (c) SBA-P (at charge density= 0.388 A/g) and in 1M H_2SO_4 electrolyte.

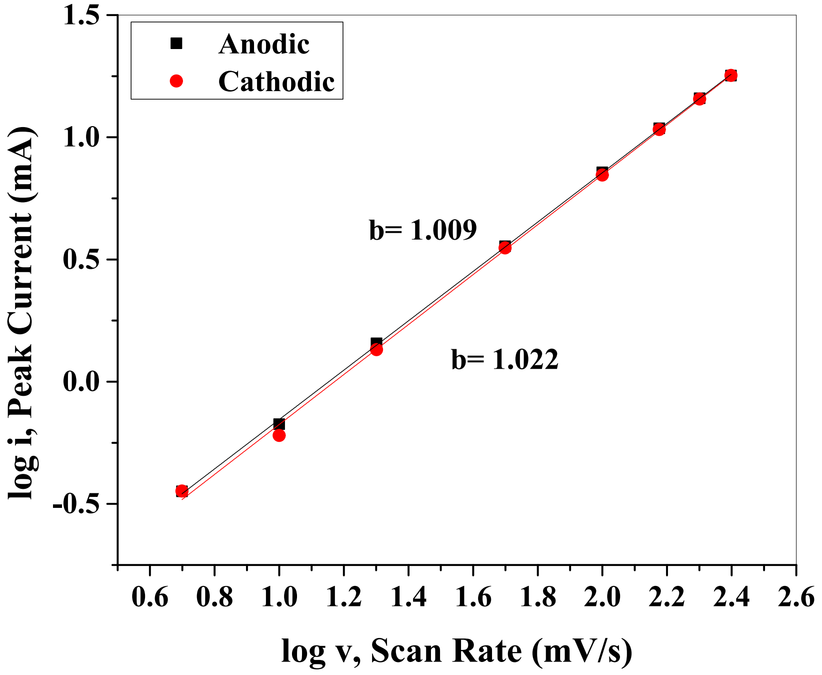

Figure S10: Plot of log (i) versus log (v) for the anodic and cathodic current peaks of mSi-P

Proposed Mechanism of Electrochemical Reaction during Charging and Discharging

Cyclic voltammograms of polyaniline (P) (Figure S4(a)) at various scan rates exhibit a redox peak which is attributed to the transition of PANI from leucoemeraldine (semiconducting state) to emeraldine (conductive form). This redox process caused pseudo capacitance of PANI. Apparently, the mSi-P composite (Figure 8(a)) has a similar electrochemical response as that of the P, but peak current of mSi-P increases greatly which implies a larger electrode capacitance. High electrochemical utilization of mSi-P is due to its larger specific surface area as compared to P and hence more electroactive sites.

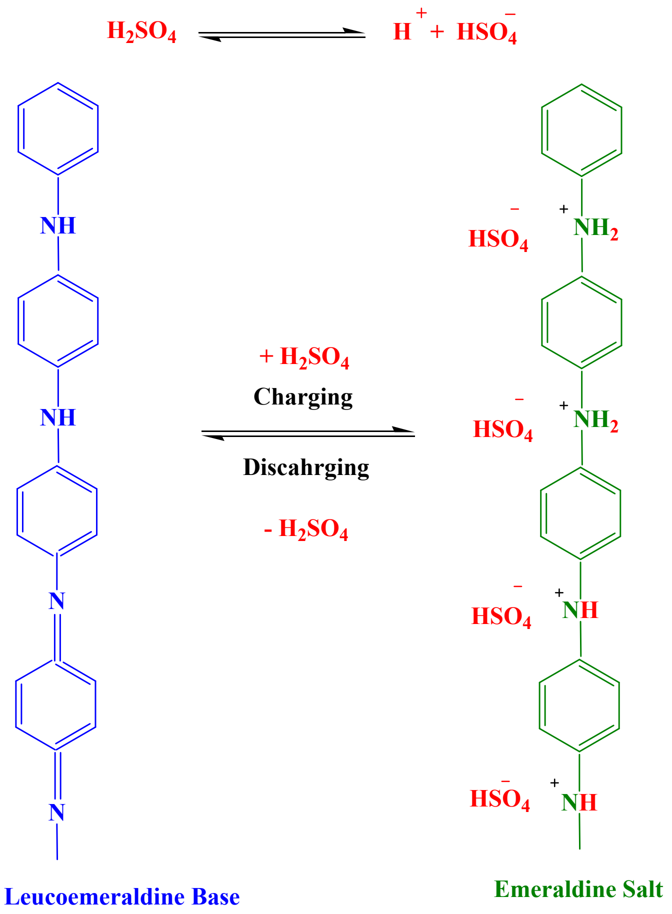

Figure S11: Proposed mechanism of electrochemical reaction during charging and discharging in 1M H_2SO_4 electrolyte.
